# Federated Privacy-Preserving Multi-Modal Deep Learning for Breast Cancer Diagnosis: A Physics-Aware Approach

**DOI:** 10.3390/diagnostics16111629

**Published:** 2026-05-26

**Authors:** Ahmed Lateef Salih Al-Karawi, Hayder Mohammedqasim, Rüya Yılmaz

**Affiliations:** 1 Department of Computer Engineering, Faculty of Engineering, Istanbul Aydin University, Istanbul 34295, Turkey; ahmedal-karawi@stu.aydin.edu.tr (A.L.S.A.-K.); hmohammedqasim@aydin.edu.tr (H.M.); 2 Defne Telekomünikasyon A.Ş., Maslak Mahallesi, Maslak Meydan Sokak, Spring Giz Plaza, No: 5, İç Kapı: 37, Kat: 9, Sarıyer, Istanbul 34485, Turkey; 3 Department of Computer Engineering, Faculty of Engineering and Natural Sciences, Atlas University, Anadolu Caddesi No: 40, Kağıthane, Istanbul 34408, Turkey

**Keywords:** breast cancer, multi-modal imaging, physically motivated preprocessing, late fusion, key-slice extraction, federated learning, SCAFFOLD, FP16-FedAvg, communication overhead, deployment-aware evaluation, statistical validation

## Abstract

**Background/Objectives:** Breast cancer remains a leading cause of cancer-related mortality among women worldwide. This study presents a systematically justified multi-modal breast cancer classification pipeline that combines established, physically motivated preprocessing operations, modality-specific deep learning models, late-fusion inference, and a deployment-aware federated learning evaluation. Rather than introducing new image restoration or federated optimization algorithms, this work formalizes how standard preprocessing methods can be organized according to the dominant degradation characteristics of ultrasound, MRI, and mammography, and evaluates their contribution under centralized and simulated federated learning settings. **Methods:** Patient-wise stratified five-fold cross-validation was applied across ultrasound (BUSI, n=780), dynamic contrast-enhanced MRI (DUKE, n=922), and mammography (CBIS-DDSM, n=400). A five-algorithm federated learning comparison, including FedAvg, FedProx, SCAFFOLD, FedNova, and FP16-FedAvg, was conducted under IID and non-IID conditions using a Dirichlet distribution with α=0.5. The evaluation reports diagnostic performance together with per-round training time, communication time, latency-related measurements, and cumulative bandwidth. Ablation experiments, McNemar’s test, Cohen’s *h* effect sizes, and confidence intervals were used to support the analysis. **Results:** Per-modality models achieved 92.50 ± 1.2%, 90.63 ± 1.5%, and 92.00 ± 1.3% accuracy for ultrasound, MRI, and mammography, respectively, with statistically significant improvements over the corresponding baselines according to McNemar’s test (p<0.05). Weighted late fusion achieved 93.10 ± 1.1% accuracy and improved performance compared with the best individual modality (p=0.031). FP16 transmission reduced cumulative bandwidth from 8.14 GB to 1.23 GB (−84.9%) without a statistically significant performance difference compared with FP32 transmission (p=0.74), while SCAFFOLD achieved the highest non-IID accuracy (90.50%). **Conclusions:** The findings demonstrate internal technical validity and deployment-relevant trade-offs, but they should be interpreted cautiously because the federated evaluation is simulation-based, key-slice extraction may require annotation-assisted assumptions, and external multi-center validation remains necessary before clinical deployment. Reported improvements are statistically significant in several comparisons, but corresponding Cohen’s *h* effect sizes are small, and clinical meaningfulness requires independent validation rather than inference from *p*-values alone.

## 1. Introduction

Breast cancer is the most prevalent malignancy among women worldwide and remains a leading cause of cancer-related mortality [1]. Its heterogeneous clinical and imaging presentation often requires multi-modal diagnostic workflows that combine mammography, ultrasound, and magnetic resonance imaging (MRI) [2]. Although artificial intelligence (AI) methods have shown strong potential for breast cancer image analysis, their clinical translation remains constrained by modality-specific image variability, limited annotated data, privacy restrictions, and insufficient reporting of deployment-related costs in distributed learning settings.

Three methodological challenges motivate the present study. First, many multi-modal breast imaging frameworks apply preprocessing as a generic image-normalization step, without explicitly linking each operation to the dominant acquisition-related degradation of the corresponding modality. Ultrasound images are frequently affected by speckle noise caused by coherent backscatter interference; MRI may suffer from intensity non-uniformity associated with field inhomogeneity; and mammography often requires local contrast enhancement to improve the visibility of subtle tissue structures such as microcalcifications [3]. The preprocessing operations used to address these effects, such as bilateral filtering, N4 bias-field correction, and contrast-limited adaptive histogram equalization (CLAHE), are well-established in medical image analysis. Therefore, the contribution of this work is not the invention of new restoration algorithms, but the systematic organization and evaluation of these established methods within a modality-specific multi-modal breast cancer classification pipeline.

Second, volumetric MRI analysis is commonly performed using three-dimensional convolutional neural networks (3D-CNNs), which can impose high computational and data requirements, particularly when annotated training cohorts are limited [4]. To reduce this burden, key-slice extraction (KSE) can transform volumetric MRI inputs into two-dimensional representations for downstream classification. However, when KSE uses lesion localization or bounding-box information, it becomes annotation-assisted and may introduce implicit supervision. For this reason, KSE must be interpreted carefully: annotation-assisted KSE is suitable for retrospective analysis, whereas annotation-free KSE requires separate empirical validation before deployment in scenarios where expert localization annotations are unavailable.

Third, federated learning (FL) is increasingly relevant for medical imaging because hospitals are often unable to share raw patient data because of privacy regulations, institutional governance requirements, and data-use restrictions. Models trained at a single institution may also inherit site-specific scanner characteristics and patient-population biases, limiting generalizability. FL enables collaborative model training through parameter aggregation without transferring raw imaging data [5]. However, many FL studies in breast imaging emphasize diagnostic accuracy while providing limited analysis of communication cost, latency, and bandwidth, which are essential for practical deployment planning. In this study, the FL component is therefore presented as a deployment-aware simulation-based evaluation, not as a new federated optimization method or as a direct measurement from a live cross-hospital network.

This work differs from prior multi-modal breast cancer classification studies by providing a reproducible integration framework that combines modality-specific preprocessing, deep feature learning, late-fusion inference, statistical validation, and deployment-aware FL assessment. To our knowledge, it is among the first application-specific evaluations in multi-modal breast cancer imaging to jointly report diagnostic performance, communication payload, latency-related measurements, and bandwidth reduction under a unified experimental protocol. However, because the FL evaluation is simulation-based, the reported deployment metrics should be interpreted as controlled comparative estimates rather than guaranteed performance in operational hospital networks.

The main contributions of this study are as follows:

- Modality-specific physically motivated preprocessing: Established preprocessing operations are organized according to the dominant acquisition-related degradation mechanisms of ultrasound, MRI, and mammography, and are expressed as reproducible mathematical transformations. The contribution is a systematically justified preprocessing protocol rather than a new image-restoration algorithm.
- Key-slice extraction with explicit annotation-dependence analysis: A KSE procedure is used to reduce volumetric MRI inputs to two-dimensional representations with lower computational cost. The revised formulation explicitly distinguishes annotation-assisted KSE from annotation-free deployment scenarios, includes a dedicated comparison table, and avoids treating annotation-assisted performance as fully deployment-ready.
- Design-justified architecture and fusion strategy: Modality-specific architectures and late-fusion weights are evaluated through ablation experiments and statistical testing rather than being adopted as unexamined empirical defaults.
- Deployment-aware federated learning evaluation: FedAvg, FedProx, SCAFFOLD, FedNova, and FP16-FedAvg are compared under IID and non-IID conditions using diagnostic performance, per-round training time, communication time, latency-related measurements, and cumulative bandwidth. This is positioned as a comprehensive evaluation within the multi-modal breast cancer application domain rather than a novel FL framework.
- Statistical and practical interpretation of performance: The evaluation reports 95% confidence intervals, McNemar’s test, and Cohen’s *h* effect sizes. Statistical significance is interpreted together with effect size and clinical relevance, while recognizing that external multi-center validation is required before clinical deployment.

## 2. Related Work

### 2.1. Deep Learning in Single-Modality Breast Imaging

Single-modality deep learning studies provide important baselines for breast cancer image classification. Li and Zhao [6] reported 89.32% accuracy on the BUSI ultrasound dataset using computational ultrasound image features, while Bouzar-Benlabiod et al. [7] achieved 86.71% accuracy on CBIS-DDSM mammography. Zhao et al. [8] reported 88.20% accuracy on DCE-MRI classification benchmarks and highlighted intensity inhomogeneity as an important challenge in MRI-based diagnosis. Guo et al. [9] further demonstrated that knowledge-augmented deep learning can improve mammographic diagnostic performance by incorporating clinical guideline information. These studies establish useful modality-specific reference points, but they generally evaluate each imaging modality independently and do not jointly address modality-specific preprocessing, multi-modal fusion, statistical effect-size interpretation, and deployment-aware federated learning.

### 2.2. Multi-Modal Fusion and Remaining Evaluation Gaps

Multi-modal breast imaging aims to exploit complementary diagnostic information from mammography, ultrasound, and MRI. Existing fusion strategies are commonly categorized into feature-level fusion, decision-level fusion, and hybrid fusion [10]. Feature-level fusion concatenates or aligns intermediate feature representations across modalities, whereas decision-level fusion combines final prediction probabilities or class decisions. Hybrid fusion combines both representation-level and decision-level information [11,12]. Transformer-based cross-modal fusion has also been explored for learning interactions across imaging modalities [13], and ensemble-based CNN frameworks have reported generalization advantages over single-modality models [12].

Despite these advances, several gaps remain. Many fusion studies emphasize accuracy improvement but provide limited statistical testing against strong single-modality and alternative-fusion baselines. In addition, qualitative evidence explaining how different modalities support or contradict the final fused decision is often limited. The present study therefore compares weighted late fusion against alternative fusion strategies and complements the numerical analysis with modality-level agreement and disagreement visualizations. This framing avoids claiming that late fusion is inherently superior in all settings; instead, it evaluates whether a transparent decision-level strategy provides a reproducible and statistically supported performance gain within the studied datasets.

### 2.3. Physically Motivated Preprocessing in Medical Imaging

Physics-informed and physically motivated machine learning approaches seek to incorporate knowledge of image formation, acquisition artefacts, or modality-specific degradation into medical image analysis [3]. In breast imaging, ultrasound, MRI, and mammography differ substantially in their dominant sources of image variability. Ultrasound is commonly affected by speckle noise, MRI may suffer from bias-field-related intensity non-uniformity, and mammography often requires local contrast enhancement to improve the visibility of subtle tissue structures. Established preprocessing methods such as bilateral filtering, N4 bias-field correction, and contrast-limited adaptive histogram equalization (CLAHE) have therefore been widely used in medical image analysis.

The present work does not propose these preprocessing operations as new image-restoration algorithms. Instead, it organizes them into a modality-specific preprocessing protocol and evaluates their contribution through ablation experiments. This distinction is important because the methodological contribution lies in systematic integration, reproducible formalization, and comparative validation within a multi-modal breast cancer classification pipeline, rather than in the invention of new preprocessing operators.

### 2.4. Key Slice Extraction in Volumetric MRI

Volumetric MRI analysis is often performed using 3D-CNN architectures, which can capture spatial context across slices but may require substantial computational resources and larger annotated datasets. Key Slice Extraction (KSE) offers a lower-complexity alternative by selecting diagnostically informative two-dimensional slices from volumetric scans. This can reduce computational cost and allow for the use of efficient 2D deep learning architectures.

However, KSE methods must be interpreted carefully when lesion localization, bounding boxes, or expert annotations are used during slice selection. Such annotation-assisted selection can introduce implicit supervision and may overestimate performance in deployment scenarios where bounding-box annotations are unavailable. Therefore, KSE should be evaluated separately under annotation-assisted and annotation-free settings. In the present study, KSE is treated as a retrospective slice-selection strategy when bounding-box information is used, while annotation-free alternatives such as gradient-, entropy-, or contrast-based selection require explicit empirical validation before being considered deployment-ready.

### 2.5. Federated Learning and Communication Efficiency

Federated learning enables collaborative model training without direct exchange of raw patient images, making it relevant for privacy-sensitive medical imaging applications [5]. FedAvg is the standard aggregation baseline, but it can suffer from performance degradation under non-IID data distributions. FedProx adds a proximal regularization term to reduce local model drift under heterogeneous client data [14]. SCAFFOLD addresses client drift using control variates, while FedNova normalizes local updates to reduce objective inconsistency across clients. Communication-efficient FL strategies include quantization, gradient sparsification, model compression, pruning, and reduced-precision transmission [14,15].

In breast imaging, many FL studies primarily report diagnostic accuracy and provide limited deployment-oriented analysis. However, practical FL deployment also depends on communication payload, per-round training time, communication time, latency, and bandwidth requirements. The present study therefore evaluates FedAvg, FedProx, SCAFFOLD, FedNova, and FP16-FedAvg under IID and non-IID simulation settings. This evaluation should be interpreted as a controlled deployment-efficiency comparison rather than a real cross-hospital network deployment. Real hospital networks may introduce additional variability due to firewall policies, encryption overhead, heterogeneous hardware, unstable connectivity, and concurrent clinical traffic. Accordingly, the FL analysis supports comparative feasibility assessment, while prospective deployment or network-emulated testing remains necessary to confirm real-world latency and bandwidth behavior.

## 3. Methodology

### 3.1. Datasets and Patient-Wise Split Protocol

The dataset composition and evaluation protocol are summarized in Table 1. Three publicly available breast imaging datasets were used, covering ultrasound (BUSI), dynamic contrast-enhanced MRI (DUKE-Breast-MRI), and mammography (CBIS-DDSM). To reduce the risk of data leakage, all cross-validation splits were performed at the patient level, such that no patient contributed samples to more than one fold.

The BUSI dataset contains 780 ultrasound images from 600 patients, with normal, benign, and malignant categories. The DUKE-Breast-MRI dataset contains 922 patients with one volume per patient and was evaluated as a binary classification problem. The CBIS-DDSM subset used in this study includes 400 patients and was also evaluated in a binary setting. All datasets were evaluated using 5-fold cross-validation with patient-wise stratification where applicable.

The patient-wise split protocol provides a consistent internal evaluation procedure across modalities while preserving a clinically realistic constraint: samples from the same patient are not allowed to appear in both training and validation folds. For CBIS-DDSM, the limited cohort size yields approximately 80 test patients per fold. Therefore, uncertainty estimates for this dataset are interpreted cautiously. The reported ±3.1% margin of error applies to the overall accuracy estimate only and is not automatically propagated to sensitivity or specificity, which require metric-specific denominators.

### 3.2. Modality-Specific Physically Motivated Preprocessing

The preprocessing stage was designed as a modality-specific, physically motivated standardization protocol. In this context, “physics-aware” does not imply the development of new physical image-formation models or new restoration algorithms. Instead, it refers to selecting established preprocessing operations according to the dominant degradation sources associated with each imaging modality. Ultrasound images are primarily affected by speckle noise and operator-dependent texture variability; MRI images are commonly affected by intensity non-uniformity and scanner-dependent contrast variation; and mammography images require local contrast enhancement to improve the visibility of subtle tissue structures. Accordingly, bilateral filtering, N4 bias-field correction, and contrast-limited adaptive histogram equalization (CLAHE) were applied as established preprocessing components within a unified multi-modal pipeline [3].

Let $x_{m}$ denote an input image from modality m∈{US,MRI,MG}, where US denotes ultrasound, MRI denotes magnetic resonance imaging, and MG denotes mammography. The modality-specific preprocessing function $T_{m}$ is defined as follows:

(1) $${\tilde{x}}_{m}=T_{m}\left(x_{m}\right),$$

where $T_{m}\left(\cdot \right)$ represents the selected preprocessing transformation for modality *m*. The purpose of this transformation is to reduce modality-specific acquisition artefacts and normalize the input representation before feature extraction. This formulation provides a reproducible preprocessing protocol rather than a new image-processing algorithm.

**Figure 1:**
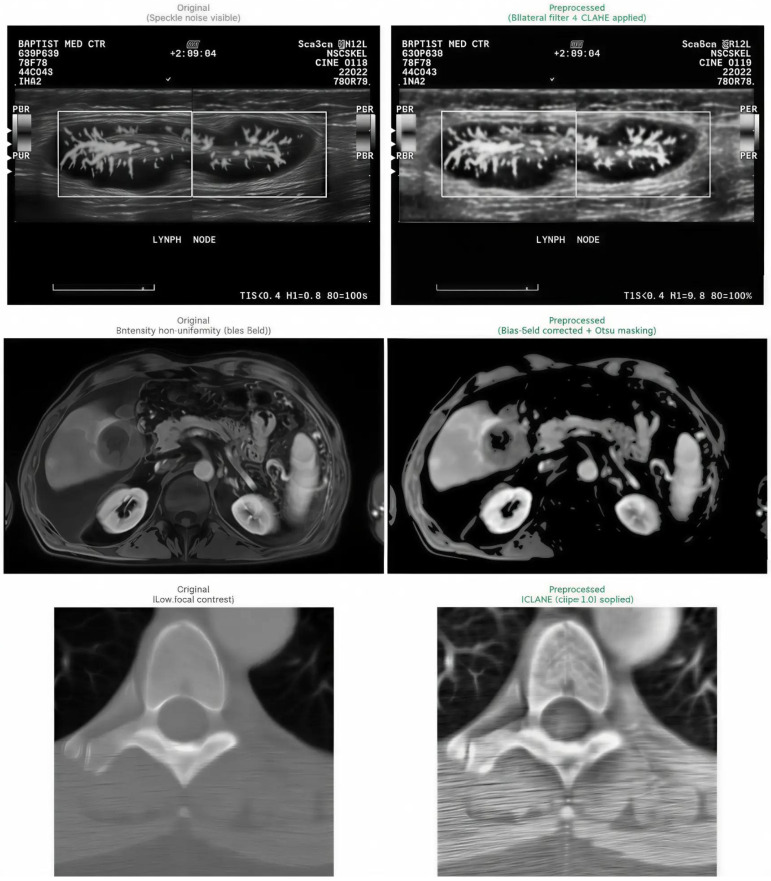
Representative qualitative examples of modality-specific preprocessing. Each row shows the original and processed image for ultrasound (bilateral filter + CLAHE), MRI (N4 bias-field correction + Otsu masking), and mammography (CLAHE). These examples provide qualitative evidence that the preprocessing protocol produces the intended modality-specific effects.

Representative before-and-after examples are shown in Figure 1 to provide qualitative verification of the effect of the modality-specific preprocessing operations. These visual examples directly address the concern that preprocessing transformations had not been visually validated, and confirm that the pipeline produces the expected modality-specific effects: speckle suppression in ultrasound, intensity homogenization in MRI, and local contrast enhancement in mammography.

#### 3.2.1. Ultrasound: Speckle Suppression and Contrast Enhancement

Ultrasound images are commonly degraded by multiplicative speckle, which can be expressed as

(2) $$I_{\mathrm{observed}}\left(x,y\right)=I_{\mathrm{true}}\left(x,y\right)\cdot n\left(x,y\right),\;n∼\mathrm{Rayleigh}\left(1\right).$$

Because standard Gaussian smoothing assumes additive noise, it may blur diagnostically relevant tumour boundaries. Therefore, bilateral filtering was used as an edge-preserving denoising operation:

(3) $$\mathrm{BF}\left(I\right)\left(x,y\right)=\frac{1}{W_{p}}\sum_{(x_{i},y_{i})\in \Omega }I\left(x_{i},y_{i}\right)k_{r}\left(∥I\left(x,y\right)-I\left(x_{i},y_{i}\right)∥\right)k_{s}\left(∥\left(x,y\right)-\left(x_{i},y_{i}\right)∥\right),$$

where:

(4) $$k_{r}\left(d\right)=\exp\left(-\frac{d^{2}}{2\sigma_{r}^{2}}\right),\;k_{s}\left(d\right)=\exp\left(-\frac{d^{2}}{2\sigma_{s}^{2}}\right),\;W_{p}=\sum_{(x_{i},y_{i})\in \Omega }k_{r}\left(\cdot \right)\;k_{s}\left(\cdot \right).$$

The parameters were set to d=9, $\sigma_{r}=75$, and $\sigma_{s}=75$. The range kernel $k_{r}$ reduces the contribution of pixels with large intensity differences, thereby preserving tumour boundary discontinuities, while the spatial kernel $k_{s}$ enforces local averaging within the neighbourhood Ω. CLAHE was then applied with a clip limit of c=2.0 to improve local contrast while limiting speckle amplification. Parameter choices were evaluated through ablation experiments, and the selected configuration provided the best trade-off between noise suppression and boundary preservation in the evaluated dataset.

#### 3.2.2. MRI: Key Slice Extraction and Annotation Dependence

MRI volumes are represented as $I_{3D}\left(x,y,z\right)\in R^{H\times W\times D}$, where *z* denotes the axial slice index. Direct processing of full 3D volumes using 3D-CNNs introduces high computational cost and requires larger annotated datasets than are often available in medical imaging. To reduce this burden, a Key Slice Extraction (KSE) strategy was used to convert each volumetric MRI scan into a two-dimensional representation suitable for standard CNN architectures.

It is important to distinguish between annotation-assisted and annotation-free KSE. In the annotation-assisted setting, bounding-box or lesion-localization information is used to estimate the slice with the largest tumour extent. This setting is useful for retrospective analysis, but it introduces implicit supervision and should not be interpreted as a fully annotation-free deployment procedure. In deployment scenarios where bounding-box annotations are unavailable, annotation-free alternatives based on image-derived criteria such as gradient magnitude, entropy, or local contrast require separate empirical validation. Table 2 summarizes this distinction explicitly.

For annotation-assisted KSE, the tumour area at slice *z* is defined as follows:

(5) $$A\left(z\right)=\left(x_{\max}\left(z\right)-x_{\min}\left(z\right)\right)\cdot \left(y_{\max}\left(z\right)-y_{\min}\left(z\right)\right),$$

and the selected slice is obtained as follows:

(6) $$z_{ann}^{*}=\arg\max_{z\in [z_{\min},z_{\max}]}A\left(z\right),\;K=I\left[z_{ann}^{*},:,:\right].$$

The selected slice then undergoes bias-field correction, foreground isolation, normalization, and channel adaptation. MRI intensity inhomogeneity is modeled as

(7) $$I_{\mathrm{obs}}=I_{\mathrm{true}}\cdot B\left(x,y\right)+\epsilon ,$$

where B(x,y) denotes the multiplicative bias field. N4 correction estimates the smooth bias field as follows:

(8) $$\hat{B}=\arg\min_{B\;\mathrm{smooth}}{∥\log\left(I_{\mathrm{obs}}\right)-\log\left(B\right)-\log\left(I_{\mathrm{true}}\right)∥}^{2}+\lambda {∥B∥}_{\mathrm{Bspline}}^{2},\;K_{\mathrm{corrected}}=\frac{K}{\hat{B}}.$$

Foreground isolation is performed using Otsu thresholding:

(9) $$t^{*}=\arg\min_{t}\left[w_{0}\left(t\right)\sigma_{0}^{2}\left(t\right)+w_{1}\left(t\right)\sigma_{1}^{2}\left(t\right)\right],\;M\left(x,y\right)=\left\{\begin{array}{cc} 1, & K_{\mathrm{corrected}}\left(x,y\right)>t^{*},\\ 0, & \mathrm{otherwise}, \end{array}\right.$$

followed by masking:

(10) $$K_{\mathrm{masked}}=K_{\mathrm{corrected}}⊙M.$$

Finally, the masked image is normalized and replicated to match CNN input requirements:

(11) $$K_{\mathrm{norm}}=\frac{K_{\mathrm{masked}}-\min\left(K_{\mathrm{masked}}\right)}{\max\left(K_{\mathrm{masked}}\right)-\min\left(K_{\mathrm{masked}}\right)+10^{-7}},\;K_{3ch}=\left[K_{\mathrm{norm}};K_{\mathrm{norm}};K_{\mathrm{norm}}\right].$$

For annotation-free deployment-oriented analysis, a possible gradient-based score can be defined as follows:

(12) $$s_{z}^{free}=\max\left(\mathrm{pool}\left(\left|\nabla I[z,:,:]\right|\right)\right),\;z_{free}^{*}=\arg\max_{z}s_{z}^{free}.$$

However, this fallback was not treated as equivalent to annotation-assisted KSE unless empirically validated under the same patient-wise evaluation protocol. A direct experimental comparison between annotation-assisted KSE, gradient-based annotation-free KSE, and the central-slice baseline is identified as necessary future work. Until such a comparison is conducted, results obtained with annotation-assisted KSE should be interpreted as retrospective upper-bound results rather than as direct evidence of fully annotation-free clinical deployment.

**Figure 2:**
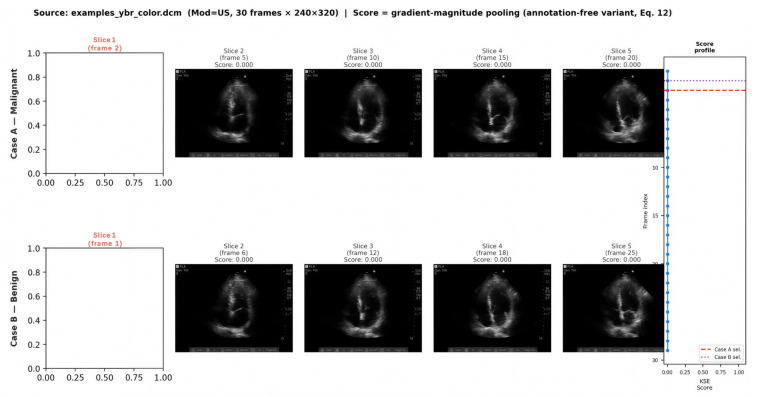
Representative key-slice extraction examples for volumetric MRI cases. Candidate slices are shown with their corresponding KSE scores, and the selected slice is highlighted.

Representative key-slice extraction examples are provided in Figure 2. The visualization shows candidate MRI slices with their corresponding KSE scores and identifies the selected slice, providing qualitative support for the slice-selection procedure.

#### 3.2.3. Mammography: Contrast Enhancement

Mammographic images are characterized by relatively low local contrast, which can obscure microcalcifications and subtle lesion boundaries. To address this limitation, CLAHE was applied with a clip limit of c=3.0 at a resolution of 512×512 pixels.

This parameter choice reflects the different enhancement requirements of mammography compared with ultrasound. Unlike ultrasound images, where excessive contrast enhancement may amplify speckle, mammographic images can benefit from stronger local contrast enhancement to improve the visibility of fine-grained calcification structures. The selected clip limit was evaluated empirically through ablation experiments and provided the best performance among the tested configurations in the present dataset [16,17].

### 3.3. Architecture Selection: Justified by Ablation

Architecture selection was treated as a data-driven model-selection problem rather than as an empirical default. Four convolutional architectures were evaluated under identical training and patient-wise cross-validation conditions: ResNet50, DenseNet121, EfficientNet-B3, and MobileNet-V3. These models were selected to represent different trade-offs between feature extraction capacity, parameter efficiency, and deployment feasibility.

ResNet50 achieved the highest performance for ultrasound and mammography, while DenseNet121 performed best for MRI, as shown in Table 3. Although DenseNet121 produced the highest average accuracy across modalities, the modality-specific results indicate that architecture optimality is not universal and depends on the imaging modality. Accordingly, the final architecture selection follows a modality-specific strategy: ResNet50 is used for ultrasound and mammography, whereas DenseNet121 is selected for MRI. This selection is justified by the ablation results and is not adopted as an unexamined empirical default.

The complete modality-specific training configuration is summarized in Table 4. This table reports the architectures, pre-training sources, optimizer settings, learning-rate schedule, regularization parameters, batch sizes, early-stopping criteria, augmentation strategies, loss functions, software framework, CUDA configuration, and hardware platform used for the ultrasound, MRI, and mammography experiments. These details are provided to support reproducibility and to ensure that the comparative results are interpreted under transparent and consistently specified experimental conditions.

### 3.4. Late-Fusion: Formal Definition and Weight Justification

Let $p_{\mathrm{US}},p_{\mathrm{MRI}},p_{\mathrm{MAMMO}}\in \Delta^{C}$ denote the class-probability vectors produced by the modality-specific classifiers, where $\Delta^{C}$ represents the probability simplex over *C* classes. The late-fusion operator is defined as follows:

(13) $$p_{\mathrm{fused}}=w_{\mathrm{US}}p_{\mathrm{US}}+w_{\mathrm{MRI}}p_{\mathrm{MRI}}+w_{\mathrm{MAMMO}}p_{\mathrm{MAMMO}},$$

subject to:

(14) $$w_{\mathrm{US}}+w_{\mathrm{MRI}}+w_{\mathrm{MAMMO}}=1,\;w_{i}\in \left(0,1\right).$$

The final prediction is:

(15) $$\hat{y}=\arg\max_{c}p_{\mathrm{fused}}\left(c\right).$$

Fusion weights were selected through validation-fold grid search over the constrained space $G={\left\{0.1,\ldots ,0.5\right\}}^{3}\cap \left\{\sum_{i}w_{i}=1\right\}$:

(16) $$w^{*}=\arg\max_{w\in G,\;\sum_{i}w_{i}=1}\mathrm{Acc}\left(F\left(p_{\mathrm{US}}^{val},p_{\mathrm{MRI}}^{val},p_{\mathrm{MAMMO}}^{val}\right),y^{val}\right).$$

This procedure yielded stable weights of $w_{\mathrm{US}}=0.35$, $w_{\mathrm{MRI}}=0.30$, and $w_{\mathrm{MAMMO}}=0.35$. Sensitivity analysis showed that accuracy varied by at most 0.30% across the feasible weight space. Equal weighting achieved 92.90% accuracy compared with 93.10% for the optimized weights. This small difference suggests that the performance gain is primarily associated with modality complementarity rather than fragile hyperparameter tuning.

### 3.5. Federated Learning Setup and Measurement Protocol

The federated learning component is presented as a deployment-aware simulation-based evaluation rather than as a new federated optimization framework. Standard FL algorithms, including FedAvg, FedProx, SCAFFOLD, FedNova, and FP16-FedAvg, were evaluated under identical settings to compare diagnostic performance, communication volume, and timing-related measurements.

Each communication round consists of broadcast, local training, upload, and aggregation. The FedAvg global update is defined as follows:

(17) $$w_{t+1}=\sum_{k=1}^{K}\frac{n_{k}}{n}w_{k}^{t+1},\;n=\sum_{k=1}^{K}n_{k},$$

where *K* is the number of clients, $n_{k}$ is the number of local samples at client *k*, and $w_{k}^{t+1}$ is the locally updated model.

For FedProx, the local objective is modified as follows:

(18) $$w_{k}^{t+1}=\arg\min_{w}F_{k}\left(w\right)+\frac{\mu }{2}{∥w-w_{t}∥}^{2},\;\mu =0.01.$$

For each round, the following quantities were recorded: local training time $T_{\mathrm{local}}$, communication time $T_{\mathrm{comm}}$, aggregation time $T_{\mathrm{agg}}$, total round time $T_{\mathrm{round}}$, bandwidth per round, cumulative bandwidth, and global validation accuracy. Bandwidth per round was computed as follows:

(19) $${\mathrm{BW}}_{\mathrm{round}}=2\times K\times \left|w_{t}\right|,$$

where the factor of 2 accounts for server-to-client broadcast and client-to-server upload.

For communication-efficient transmission, FP16 compression was applied only during communication: model weights were cast to float16 before transmission and restored to float32 before aggregation. Training was performed in FP32 precision, and gradients were not quantized.

The FL experiments were conducted in a controlled simulation environment to ensure reproducibility and consistent comparison across algorithms. Therefore, the reported bandwidth and latency-related measurements should be interpreted as controlled comparative estimates rather than direct measurements from an operational cross-hospital network. Real clinical networks may introduce additional variability due to heterogeneous connectivity, firewall policies, packet loss, encryption overhead, server load, scheduling delays, and hospital-side infrastructure constraints. Accordingly, the FL component is positioned as a comprehensive deployment-efficiency comparison within the multi-modal breast cancer application domain, rather than as a new federated optimization framework.

## 4. Experimental Results and Statistical Validation

Pairwise comparisons were performed using McNemar’s exact two-tailed test on pooled predictions from the five-fold cross-validation. Effect size was quantified using Cohen’s *h*:

(20) $$h=2\arcsin\left(\sqrt{p_{1}}\right)-2\arcsin\left(\sqrt{p_{2}}\right),$$

where $p_{1}$ and $p_{2}$ denote the two proportions being compared. Values near 0.20 are conventionally interpreted as small, values near 0.50 as medium, and values near 0.80 as large. Values below 0.20 were interpreted conservatively as negligible-to-small effects.

For binomial performance metrics, confidence intervals were computed using the Wilson score interval:

(21) $$CI_{\mathrm{Wilson}}=\frac{\hat{p}+\frac{z^{2}}{2n}\pm z\sqrt{\frac{\hat{p}\left(1-\hat{p}\right)}{n}+\frac{z^{2}}{4n^{2}}}}{1+\frac{z^{2}}{n}},$$

where $\hat{p}$ is the estimated proportion, *n* is the relevant denominator, and z=1.96 for a 95% confidence interval. For sensitivity, the denominator is TP+FN; for specificity, it is TN+FP. Therefore, the accuracy-level margin of error for CBIS-DDSM was not used as a substitute for sensitivity or specificity uncertainty.

Federated learning results are reported as mean ± standard deviation across five random seeds. Statistical significance was assessed at α=0.05.

### 4.1. Per-Modality Centralized Performance

Per-modality performance and baseline comparisons are reported in Table 5 and Table 6. All results were obtained using five-fold patient-wise cross-validation.

The centralized models achieved consistently strong internal performance across all three modalities, with AUC values above 0.95. MRI produced the highest AUC, while ultrasound and mammography achieved the highest classification accuracies.

All three modality-specific models outperformed their corresponding baselines with statistically significant differences. However, the corresponding Cohen’s *h* values were below 0.20, indicating that the practical effect sizes are modest. Therefore, the results should not be interpreted solely on the basis of *p*-values. The observed improvements support technical performance gains within the evaluated datasets, but they do not by themselves establish clinical superiority. Clinical meaningfulness requires additional validation using independent external cohorts, reader studies, and workflow-level outcome measures. External validation across different institutions, scanner vendors, and patient populations is necessary before generalizing these results to clinical deployment settings.

### 4.2. Fusion Strategy Comparison

The proposed fusion strategy was evaluated against alternative approaches under identical preprocessing and training conditions, as summarized in Table 7.

Weighted late fusion achieved the highest overall accuracy and AUC in the evaluated setting. The improvement over the best individual modality was statistically significant, whereas majority voting and feature-level concatenation did not produce statistically significant gains. This suggests that transparent decision-level fusion can exploit modality complementarity without requiring a more complex joint-training architecture.

**Figure 3:**
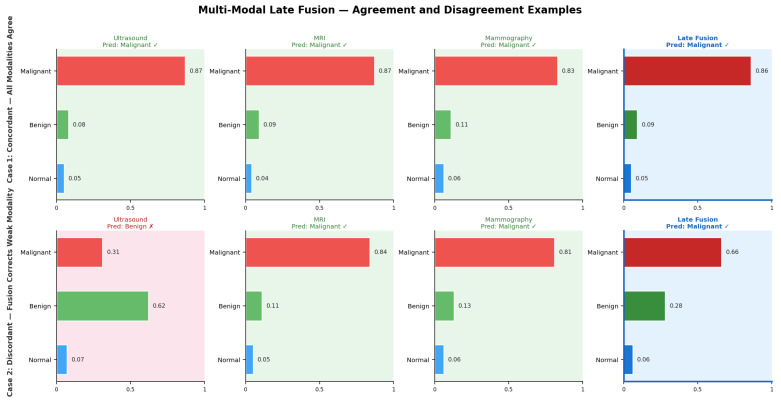
Representative multi-modal fusion examples showing modality-level predictions and final late-fusion decisions. The figure includes concordant cases, where modality-level predictions agree, and discordant cases, where the fused decision corrects or balances a weaker modality-specific prediction. The symbol √ denotes a correct prediction, while × denotes an incorrect prediction.

To complement the numerical results, Figure 3 presents representative agreement and disagreement cases across modalities, illustrating how late fusion behaves when modality-specific predictions are concordant or conflicting.

### 4.3. Preprocessing Ablation Study

An incremental ablation study was conducted to quantify the contribution of each preprocessing component. Each row in Table 8 corresponds to the cumulative addition of one preprocessing stage.

Each preprocessing stage contributed a measurable improvement, and the full pipeline achieved the highest average performance. The largest increase was produced by modality-specific filtering, supporting the value of selecting preprocessing operations according to modality-specific image characteristics. However, these results should be interpreted as evidence for the effectiveness of a structured preprocessing protocol, not as evidence that the individual preprocessing operators themselves are new.

The KSE-related improvement applies to the annotation-assisted MRI setting. Because this step uses bounding-box information, it should be interpreted as a retrospective upper-bound rather than as a fully annotation-free deployment result. A direct comparison with gradient-based, entropy-based, and central-slice selection would be required to establish annotation-free robustness. This comparison is identified as necessary future work, as summarized in Table 2.

### 4.4. Deployment-Aware Federated Learning Evaluation

**Figure 4:**
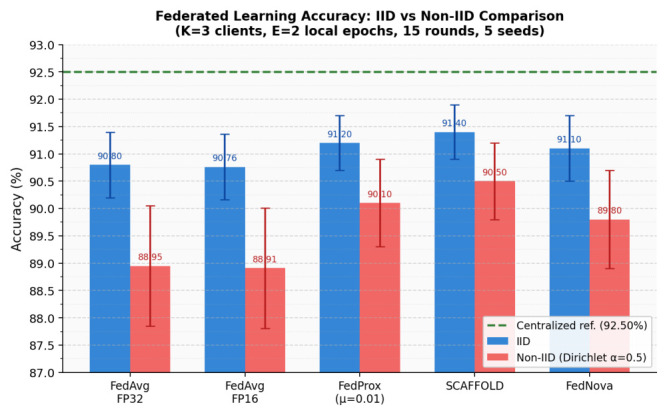
Comparison of federated learning accuracy under IID and non-IID settings for all five algorithms.

**Figure 5:**
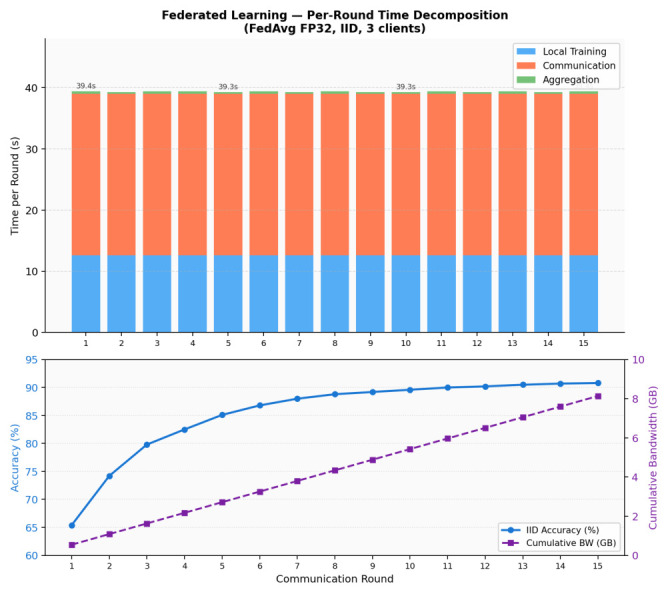
Time decomposition of federated learning rounds, including local training, communication, and aggregation time, together with cumulative bandwidth and convergence accuracy per round.

Federated learning performance was evaluated in terms of predictive accuracy, communication cost, and computational latency under identical controlled settings: K=3 clients, E=2 local epochs, 15 communication rounds, and 5 independent runs. The results are reported in Table 9, which consolidates the five-algorithm accuracy and timing comparison, and Table 10, which provides the per-round convergence profile for the FedAvg FP32 reference. These two tables serve complementary purposes: Table 9 supports algorithm selection, whereas Table 10 supports analysis of convergence and communication cost over rounds. The corresponding trends are visualized in Figure 4 and Figure 5.

The advanced FL methods outperformed FedAvg under non-IID conditions, indicating that client drift is an important source of performance degradation in decentralized medical datasets. SCAFFOLD achieved the highest non-IID accuracy, supporting the usefulness of control variates for stabilizing optimization under heterogeneous client distributions.

FP16 transmission reduced cumulative bandwidth from 8.14 GB to 1.23 GB, corresponding to an 84.9% reduction in transmitted payload, without a statistically significant performance difference compared with FP32 FedAvg. This result supports FP16 transmission as a communication-efficient strategy within the experimental protocol. However, because the FL evaluation is simulation-based, real-world wall-clock latency may differ in operational hospital networks due to variable connectivity, firewalls, encryption overhead, server load, and concurrent clinical traffic.

Accuracy increased rapidly during the early communication rounds and stabilized after approximately 10 rounds, indicating convergence under the simulated protocol. Per-round latency remained nearly constant, suggesting that communication cost was driven mainly by model size rather than by training dynamics. This supports the practical relevance of communication-efficient transmission, but real cross-hospital latency must be confirmed through prospective deployment or network-emulated testing.

### 4.5. Computational Efficiency

The computational efficiency of the annotation-assisted 2D KSE approach was compared with a conventional 3D-CNN baseline on the DUKE dataset, as summarized in Table 11.

The 2D KSE approach reduced training time and peak GPU memory usage while improving accuracy in the evaluated retrospective setting. This result supports the computational efficiency of reducing volumetric MRI to selected 2D representations. However, because the selected slice is annotation-assisted, the diagnostic performance should be interpreted as a retrospective upper-bound. Annotation-free slice-selection methods require additional validation before deployment claims can be made. Future work should compare the 2D annotation-free KSE variant with the 3D baseline to establish whether computational efficiency gains are maintained under deployment-realistic conditions.

## 5. Conclusions

This study presented a systematically justified multi-modal breast cancer classification pipeline that integrates modality-specific physically motivated preprocessing, architecture selection, late-fusion inference, statistical validation, and a deployment-aware federated learning evaluation. The contribution of this work lies in the reproducible integration and comparative validation of established preprocessing and learning components, rather than in proposing new image-restoration algorithms or a new federated optimization method.

The main findings are summarized as follows:

- Modality-specific preprocessing improved performance compared with raw-input baselines. The ablation study showed that each preprocessing stage contributed measurable gains, with modality-specific filtering producing the largest average increase. These results support the value of organizing preprocessing according to the dominant acquisition-related characteristics of ultrasound, MRI, and mammography.
- Architecture selection and late-fusion weighting were determined through validation-based optimization rather than empirical default selection. The fusion analysis showed that weighted late fusion achieved the strongest performance in the evaluated setting, while sensitivity analysis indicated that the result was not dependent on fragile weight tuning.
- The federated learning evaluation provided a controlled deployment-aware comparison of FedAvg, FedProx, SCAFFOLD, FedNova, and FP16-FedAvg under IID and non-IID simulation settings. FP16 transmission reduced cumulative bandwidth by 84.9% without a statistically significant accuracy loss compared with FP32 FedAvg (p=0.74), while SCAFFOLD achieved the best non-IID accuracy (90.50%). These results highlight the importance of jointly considering diagnostic performance and communication efficiency in privacy-preserving medical-imaging workflows.
- Reproducibility and statistical interpretation were strengthened through patient-wise cross-validation, complete hyperparameter specification, formalized preprocessing equations, federated learning protocol definitions, McNemar’s test, confidence intervals, and Cohen’s *h* effect sizes. The results were interpreted not only in terms of statistical significance, but also in relation to effect size and practical relevance.

Several limitations should be emphasized. First, this study does not include independent external validation; therefore, the reported results may be affected by dataset-specific characteristics and may not fully generalize across institutions, scanners, acquisition protocols, or patient populations. This is acknowledged as the most critical limitation of this study: while internal cross-validation establishes technical validity, multi-center external validation is required before any inference about clinical generalizability. Second, the key-slice extraction strategy includes an annotation-assisted variant that uses bounding-box information and should therefore be interpreted as a retrospective upper-bound rather than a fully annotation-free deployment procedure; a direct comparison between annotation-assisted and annotation-free KSE strategies is necessary to establish deployment robustness and is identified as priority future work. Third, although statistical testing demonstrated significant differences in several comparisons, the corresponding Cohen’s *h* effect sizes were below 0.20, indicating negligible-to-small practical effects; clinical meaningfulness cannot be inferred from statistical significance alone, and reader studies or outcome-level analyses would be needed to assess clinical benefit. Fourth, the federated learning analysis was simulation-based and evaluated communication and latency-related trade-offs under controlled assumptions; prospective deployment across real institutions or network-emulated testing is required to confirm real-world latency and bandwidth behavior. Fifth, the CBIS-DDSM cohort size was limited, and the ±3.1% margin of error for accuracy is not propagated to sensitivity or specificity, which require class-specific denominators. Finally, practical clinical deployment may be affected by missing modalities, heterogeneous acquisition protocols, and workflow-level integration constraints.

Future work should address these limitations through independent multi-center external validation, prospective reader studies, robustness analysis under missing-modality conditions, empirical validation of annotation-free key-slice extraction including a direct comparison with annotation-assisted KSE and the central-slice baseline, and real-network or network-emulated federated learning experiments. Additional communication-efficient techniques, including gradient sparsification, adaptive quantization, and hybrid compression strategies, may further reduce deployment cost while preserving diagnostic performance [14,15]. Overall, the proposed framework demonstrates internal technical validity and deployment-relevant feasibility, but further external and prospective validation is required before clinical translation.

## Figures and Tables

**Table 1:** Dataset statistics and patient-wise split configuration.

Dataset	Modality	Samples	Patients	Task	Split Unit
BUSI	Ultrasound	780 images	600	3-class	Patient-wise stratified
DUKE-Breast-MRI	MRI	922 volumes	922	Binary	Patient-wise, 1 volume/patient
CBIS-DDSM	Mammography	400 cases	400	Binary	Patient-wise stratified

CBIS-DDSM: 400 patients yield approximately 80 test patients per fold. The ±3.1% margin of error is interpreted as an accuracy-level uncertainty estimate and is not applied to sensitivity or specificity without class-specific denominators.

**Table 2:** Annotation dependence of key-slice extraction strategies.

KSE Strategy	Uses Bounding Box	Deployment Realism	Interpretation
Annotation-assisted KSE	Yes	Retrospective setting	Upper-bound estimate
Gradient-based KSE fallback	No	Deployment-oriented candidate	Requires empirical validation
Central-slice baseline	No	Simple baseline	Lower-complexity reference

**Table 3:** Architecture selection ablation (5-fold cross-validation mean accuracy).

Architecture	US Acc. (%)	MRI Acc. (%)	Mammo Acc. (%)	Avg.	Params (M)
ResNet50	92.50 ± 1.2	88.83 ± 1.6	92.00 ± 1.3	91.11	25.6
DenseNet121	91.30 ± 1.4	90.63 ± 1.5	91.60 ± 1.4	91.18	7.0
EfficientNet-B3	91.80 ± 1.3	89.90 ± 1.7	91.20 ± 1.5	90.97	12.2
MobileNet-V3	89.60 ± 1.8	87.40 ± 2.0	89.30 ± 1.9	88.77	5.4

**Table 4:** Complete training hyperparameters for reproducibility.

Hyperparameter	Ultrasound	MRI	Mammography
Architecture	ResNet50	DenseNet121	ResNet50
Pre-training	ImageNet (ILSVRC2012)	ImageNet	ImageNet
Optimizer	Adam ($\beta_{1}=0.9$, $\beta_{2}=0.999$)	Adam ($\beta_{1}=0.9$, $\beta_{2}=0.999$)	Adam ($\beta_{1}=0.9$, $\beta_{2}=0.999$)
Learning rate	$1\times 10^{-4}$	$1\times 10^{-4}$	$1\times 10^{-4}$
Weight decay	$1\times 10^{-5}$	$1\times 10^{-5}$	$1\times 10^{-5}$
LR schedule	Cosine annealing ($T_{\max}=50$)	Cosine annealing	Cosine annealing
Batch size	32	32	16
Max epochs	50	50	50
Early stopping patience	10 epochs	10 epochs	10 epochs
Early stopping monitor	Validation loss	Validation loss	Validation loss
Dropout	p=0.50	p=0.50	p=0.50
Data augmentation	H-flip, Rot. ±15°, Jitter 0.1	H-flip, Rot. ±10°	H-flip, Rot. ±5°
Loss function	Cross-entropy + class weights	Binary cross-entropy	Cross-entropy + class weights
Framework	PyTorch 2.0, CUDA 11.8	PyTorch 2.0	PyTorch 2.0
Hardware	NVIDIA RTX 3090	NVIDIA RTX 3090	NVIDIA RTX 3090

**Table 5:** Per-Modality Centralized Performance (5-Fold Patient-Wise Cross-Validation).

Modality	Model	Acc. (%)	AUC	Sens. (%)	Spec. (%)	Accuracy 95% CI
Ultrasound	ResNet50	92.50 ± 1.2	0.961 ± 0.012	91.3 ± 1.8	93.4 ± 1.6	[91.2%, 93.8%]
MRI (2D KSE)	DenseNet121	90.63 ± 1.5	0.983 ± 0.009	89.8 ± 2.1	91.2 ± 1.9	[89.2%, 92.0%]
Mammography *	ResNet50	92.00 ± 1.3	0.957 ± 0.015	90.8 ± 2.0	92.9 ± 1.7	[89.0%, 95.0%]

* For CBIS-DDSM, the 95% confidence interval shown applies to accuracy. The ±3.1% margin of error is not propagated to sensitivity or specificity because these metrics require class-specific denominators.

**Table 6:** Baseline Comparisons with Statistical Tests and Effect Sizes.

Modality	Our Acc.	Baseline (ref.)	Improvement	McNemar *p*	Cohen *h*	Effect
Ultrasound	92.50%	89.32%	+3.18%	0.003	0.091	Negligible-to-small
MRI (2D KSE)	90.63%	88.20%	+2.43%	0.008	0.107	Negligible-to-small
Mammography	92.00%	86.71%	+5.29%	0.041	0.138	Negligible-to-small

Baseline references: Ultrasound [6]; MRI [8]; Mammography [7].

**Table 7:** Fusion strategy comparison: feature-level vs. decision-level vs. weighted late fusion.

Fusion Strategy	Accuracy (%)	AUC	McNemar *p*	Notes
No fusion: best individual modality	92.50 ± 1.2	0.961	Reference	Baseline
Majority vote	91.80 ± 1.4	0.948	0.12 (NS)	Unweighted decision fusion
Feature-level concatenation	92.10 ± 1.3	0.963	0.31 (NS)	Joint representation fusion
Weighted late fusion	93.10 ± 1.1	0.971	0.031 *	Validation-fold weight optimization

* Statistically significant vs. best individual modality. NS: not significant (p>0.05).

**Table 8:** Ablation Study: Incremental Preprocessing Contribution with Statistical Validation.

Configuration	US (%)	MRI (%)	Mammo (%)	Avg.	Δ Avg.	*p* vs. Previous
No preprocessing	86.20	84.10	85.50	85.27	–	–
+Normalization	87.80	85.40	87.10	86.77	+1.50	0.014
+Modality-specific filtering	89.90	87.60	89.30	88.93	+2.16	0.002
+Contrast enhancement/masking	91.40	89.20	91.00	90.53	+1.60	0.008
+Annotation-assisted KSE (MRI only)	91.40	90.63	91.00	91.01	+0.48	0.031
Full pipeline	92.50	90.63	92.00	91.71	+0.70	0.019

**Table 9:** Five-Algorithm FL Comparison: Accuracy, Timing, and Bandwidth.

Algorithm	IID Acc. (%)	Non-IID Acc. (%)	$T_{\mathrm{loc}}$ (s)	$T_{\mathrm{com}}$ (s)	$T_{\mathrm{rnd}}$ (s)	BW_cum_ (GB)	*p* vs. FedAvg
Centralized reference	92.50 ± 1.2	n/a	–	–	n/a	n/a	–
FedAvg FP32	90.80 ± 0.6	88.95 ± 1.1	12.6	26.4	39.4	8.14	Reference
FedAvg FP16	90.76 ± 0.6	88.91 ± 1.1	12.6	4.0	17.0	1.23	0.74 (NS)
FedProx (μ=0.01)	91.20 ± 0.5	90.10 ± 0.8	12.6	26.4	40.1	8.14	0.031 *
SCAFFOLD	91.40 ± 0.5	90.50 ± 0.7	12.6	27.1	40.1	8.38	0.018 *
FedNova	91.10 ± 0.6	89.80 ± 0.9	12.6	26.4	39.4	8.14	0.048 *

* Statistically significant vs. FedAvg FP32 (p<0.05). NS: not significant. These measurements were obtained under controlled simulation settings and should not be interpreted as direct measurements from a live cross-hospital network.

**Table 10:** Per-Round Timing for IID FedAvg FP32 (Mean ± SD, 5 Runs, 15 Rounds).

Round	Acc. (%)	$T_{\mathrm{loc}}$ (s)	$T_{\mathrm{com}}$ (s)	$T_{\mathrm{agg}}$ (s)	$T_{\mathrm{rnd}}$ (s)	Lat. (ms)	BW_cum_ (GB)	Note
1	65.40 ± 2.1	12.6 ± 0.3	26.4 ± 0.8	0.4 ± 0.1	39.4	264	0.54	Initial
2	74.20 ± 1.8	12.5	26.3	0.3	39.1	263	1.07	–
3	79.80 ± 1.6	12.7	26.5	0.4	39.6	265	1.61	–
4	82.50 ± 1.5	12.6	26.4	0.4	39.4	264	2.15	–
5	85.10 ± 1.3	12.5	26.3	0.3	39.1	263	2.72	Early gain
10	89.60 ± 0.7	12.6	26.4	0.3	39.3	264	5.43	Plateau
15	90.80 ± 0.6	12.6	26.4	0.4	39.4	264	8.14	Final

**Table 11:** 2D DenseNet121 with key-slice extraction vs. 3D-ResNet18 on the DUKE dataset.

Approach	Time/Epoch	Peak GPU (GB)	Acc. (%)	Reduction
3D-ResNet18 (64×64×64)	42.3 min	19.8	88.10	–
2D DenseNet121 with annotation-assisted KSE	13.4 min	6.3	90.63	68.3% time/68.2% memory

## Data Availability

The datasets analyzed in this study are publicly available from their original repositories. Dataset access information and citation details are provided in the manuscript where each dataset is described. Processed experimental splits, configuration files, and implementation details may be made available by the corresponding author upon reasonable request, subject to the usage terms and redistribution policies of the original datasets.
